# Production of free fatty acids from various carbon sources by *Ogataea polymorpha*

**DOI:** 10.1186/s40643-022-00566-8

**Published:** 2022-07-30

**Authors:** Yunxia Li, XiaoXin Zhai, Wei Yu, Dao Feng, Aamer Ali Shah, Jiaoqi Gao, Yongjin J. Zhou

**Affiliations:** 1 Division of Biotechnology, Dalian Institute of Chemical Physics, Chinese Academy of Sciences, 457 Zhongshan Road, Dalian, 116023 People’s Republic of China; 2 CAS Key Laboratory of Separation Science for Analytical Chemistry, Dalian Institute of Chemical Physics, Chinese Academy of Sciences, Dalian, 116023 People’s Republic of China; 3 Dalian Key Laboratory of Energy Biotechnology, Dalian Institute of Chemical Physics, CAS, 457 Zhongshan Road, Dalian, 116023 People’s Republic of China; 4 University of Chinese Academy of Sciences, Beijing, 100049 People’s Republic of China; 5 Department of Microbiology, Faculty of Biological Sciences, Quaid-I-Azam University, Islamabad, 45320 Pakistan

**Keywords:** *Ogataea polymorpha*, Fatty acid, Lignocellulose, Methanol, Fermentation optimization

## Abstract

Energy shortage and environmental concern urgently require establishing the feasible bio-refinery process from various feedstocks. The methylotrophic yeast *Ogataea polymorpha* is thermo-tolerant and can utilize various carbon sources, such as glucose, xylose and methanol, which makes it a promising host for bio-manufacturing. Here, we explored the capacity of *O. polymorpha* for overproduction of free fatty acids (FFAs) from multiple substrates. The engineered yeast produced 674 mg/L FFA from 20 g/L glucose in shake flask and could sequentially utilize the mixture of glucose and xylose. However, the FFA producing strain failed to survive in sole methanol and supplementing co-substrate xylose promoted methanol metabolism. A synergistic utilization of xylose and methanol was observed in the FFA producing strain. Finally, a mixture of glucose, xylose and methanol was evaluated for FFA production (1.2 g/L). This study showed that *O. polymorpha* is an ideal host for chemical production from various carbon sources.

**Graphical Abstract:**

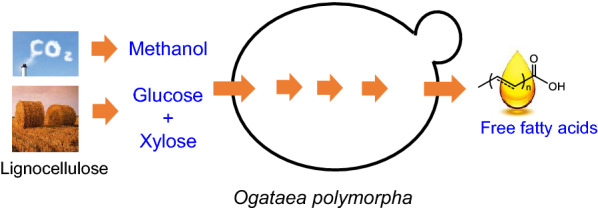

## Introduction

Fossil fuel crisis and environmental concern urgently require bio-manufacturing from sustainable, clean, and cheap feedstocks (Liu et al. 2021; Zhou et al. 2018). Lignocellulosic biomass represents for the most abundant and evenly distributed feedstock (Alonso et al. 2017), and numerous studies have explored the utilization of biomass derived sugars (glucose or xylose) as the substrate for bio-productions (Reshmy et al. 2022; Sun et al. 2021). In addition, one carbon resources such as methanol can be massively produced from natural gas, coal, biomass, and even CO_2_, which makes it an ideal feedstock for bio-manufacturing and have “carbon zero” footprint (Clomburg et al. 2017). In particular, it has been shown that methanol contributed to the pretreatment of lignocellulosic biomass (Warner et al. 2014). Therefore, co-utilization of mixed substrates, such as glucose, xylose and methanol, may be a promising route for production of chemicals and biofuels.

Free fatty acids (FFAs) have been widely used for production of detergents, lubricants, cosmetics (Tee et al. 2014) and advanced biofuels (Moore et al. 2017). However, the traditional production of FFAs and its derivatives from natural resources and chemical process cannot meet the increasing market demand, which greatly threatens the biodiversity and ecological environment (Fillet and Adrio 2016). Alternatively, microbial production is considered as a sustainable process and has attracted extensive attentions in recent years (Yu et al. 2018). Currently, engineering the central metabolism and fatty acid biosynthetic pathway enabled high-level production of FFA in various microorganisms, such as *Escherichia coli* (Xu et al. 2013), *Rhodococcus opacus* (Kim et al. 2019), *Yarrowia lipolytic* (Ledesma-Amaro et al. 2016) and *Saccharomyces cerevisiae* (Zhou et al. 2016). However, most studies obtained FFA from glucose, and co-utilization of multiple substrates for FFA production requires superior microbial chassis.

The methylotrophic yeast *Ogataea polymorpha* possesses the capacity to utilize numerous carbon sources including glucose, xylose and methanol, and the thermo-tolerant characteristic also makes it an ideal chassis organism to covert methanol and lignocellulosic materials into value-added products. Although this industrial yeast has been widely used for production of proteins (Cai et al. 2019; Ryabova et al. 2003), there is limited progress on engineering *O. polymorpha* for production of small molecules.

In this study, we explored to engineer *O. polymorpha* for production of FFA from various carbon sources using the established genetic engineering platform (Gao et al. 2021; Yu et al. 2021). The co-feeding of these carbon sources may eventually lay a foundation of *O. polymorpha* for biosynthesis of FFA and its derivatives from methanol and lignocellulosic materials.

## Materials and methods

### Strains and cultivation conditions

All strains used in this study were listed in Table1. *O. polymopha* NCYC 495 *leu1.1* was purchased from China General Microbiological Culture Collection Center (CGMCC). FD07, FD09 were constructed in this study via the CRISPR/Cas9 tool, and gRNA plasmids, donor DNA, transformation, colony verification, and selective marker removal were all based on previous methods (Gao et al., 2021). Delft minimum medium (contains 2.5 g (NH_4_)_2_SO_4_, 14.4 g KH_2_PO_4_, 0.5 g MgSO_4_•7H_2_O, 1 mL Vitamin solution and 2 mL Trace metal solution (Verduyn et al. 1992) per liter, pH value 5.6) was used for cell cultivation with glucose, xylose or methanol as carbon sources. Culturing strain 495–3 needs to add leucine (60 mg/L) in medium. All strains were cultivated in 100 mL shake flasks with a working volume of 20 mL at 37 °C, 220 rpm in a shake incubator (Zhichu ZQZY-CS8, Shanghai, China). YPD medium (20 g/L glucose, 20 g/L peptone and 10 g/L yeast extract) with a working volume of 10 mL was used to pre-culture in 50 mL tubes when methanol was used as carbon source. The pre-culture (centrifugation at 1000 × *g* for 5 min) was washed with Delft medium without any carbon source and was then re-suspended before inoculation. The same Delft medium (20 g/L glucose and 10 g/L xylose as carbon sources) were used in pre-culture with 2 mL in 15 mL tubes. The initial OD_600_ of inoculation in this study was 0.2.

**Table 1:** Strains used in this study

Strains	Genotype
495–3	*MATa; leu1.1;* (P_*GAP*_-*hCAS9*-T_*AOX1*_)
FD07	*MATa; leu1.1*:: *OPLEU2*; (P_*GAP*_-*hCAS9*-T_*AOX1*_)
FD09	*MATa; leu1.1*:: *OPLEU2*; (P_*GAP*_-*hCAS9*-T_*AOX1*_); *faa1*Δ

### Growth curve measurement

Fermentation samples were taken every 24 h during fermentation, and at the beginning of the fermentation, samples were taken every 6 or 12 h when glucose was used as the carbon source. In this study, biomass was represented by optical density at 600 nm (OD_600_), which can be converted by dry cell weight (DCW, g/L) with a coefficient of 0.2. The OD_600_ of the sample was detected by UV spectrophotometer (Macy UV-1100). Each data was displayed as mean ± standard deviation of three or four independent samples.

### FFA quantification

Quantification of FFA was performed by GC (Thermo Fisher Trace 1300), equipped with a Zebron ZB-5MS GUARDIAN capillary column (30 m* 0.25 mm* 0.25 μm, Phenomenex) and FID detector. Extraction and quantification were performed as previously reported (Zhou et al. 2016) with some modifications. The cell culture was diluted by 10 times if there were FFA pallets. 100 μL H_2_O was added to 100 μL (diluted) cell culture for dilution and then 10 μL 40% tetrabutylammonium hydroxide solution and 200 μL methylation reagent (1.245 mL methyl iodide and 1 mL pentadecanoic acid were added to 97.75 mL dichloromethane) were added. The mixtures were shaken for 30 min at 1600 rpm using a vortex mixer, and then centrifuged at 2000 × *g* for 10 min to promote phase separation. Dichloromethane layer (about 150 μL) was transferred into GC sample bottle and placed in fume cupboard until volatilization completed and then 200 μL hexane was added to resuspend the extracted methyl esters. GC program was set as follows: initial temperature of 40 °C, hold for 2 min; ramp to 180 °C at a rate of 30 °C/min; then raised to 200 °C at a rate of 40 °C/min, hold for 1 min; finally raised to 240 °C at a rate of 2 °C/min, hold for 10 min. The temperature of inlet and detection were kept at 250 °C. The injection volume was 1 μL and the flow rate of carrier gas (nitrogen) was set as 1.0 mL/min.

### Quantification of sugars and methanol

Glucose, xylose and methanol in medium were determined by HPLC (Agilent Infinity II) with 1260 RID detector (G7162A). In detail, 1.5 mL cell culture was centrifuged at 12 000 × *g* for 10 min. 700–800 μL supernatant was filtered through a 0.2 μm syringe filter, and then transferred into HPLC sample bottle. The column was eluted with 5 mM H_2_SO_4_ at a flow rate of 1 mL/min at 50 °C for 25 min. The injection volume was 20 μL.

### Statistics

Statistical analysis is performed in Office Excel Software using two-tailed *t* test method of variance ANOVA hypothesis. Significant differences are marked as *p < 0.05, **p < 0.01, and ***p < 0.001. All data are presented as mean ± s.e.m. The number of biologically independent samples for each panel is three or four.

## Results

### Engineering *O. polymorpha* for overproducing FFA

**Fig. 1:**
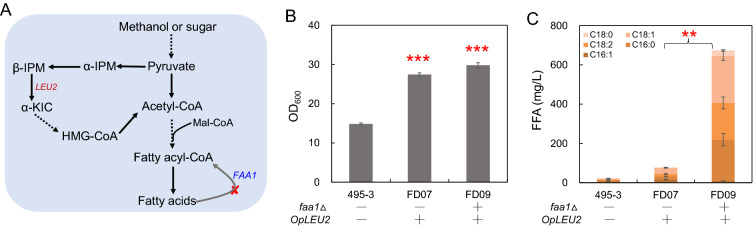
Fatty acids production from glucose in engineered *O. polymorpha*. **A** Schematic diagram of FFA synthesis pathway and leucine (LEU) metabolism in *O. polymorpha*. Deletion of fatty acyl-CoA synthetase (encoded by *FAA1*) may block the β-oxidation of FFA, which eventually result in FFA accumulation. α-IPM: α-isopropyl malic acid, β-IPM: β-isopropyl malic acid, α-KIC: α-Ketoisocaproic acid, HMG-CoA: Hydroxymeglutaryl-CoA. **B** Cell growth of *O. polymorpha* strains with integrated *OpLEU2 *in situ and disrupted *FAA1*. **C** FFA titers of *O. polymorpha* strains with integrated *OpLEU2 *in situ and disrupted *FAA1*. The fermentation was carried out in minimal medium containing 20 g/L glucose at 37 °C 220 rpm for 96 h. Data are presented as mean ± s.e.m. (n = 3 biologically independent samples). Red asterisks indicate statistical significance as determined by paired *t* test (**P < 0.01; ***P < 0.001)

The starting strain 495–3 with a copy of integrated *CAS9* gene possessed the leucine auxotroph for genetic manipulation (Gao et al., 2021). However, this auxotroph is inferred to greatly hinder cell growth, which may decrease the corresponding FFA production owing to the possible relationship with acetyl-CoA metabolism (Fig. 1A). Therefore, native gene *OpLEU2* was integrated* in situ* in strain 495–3, and the obtained strain FD07 increased the maximum OD_600_ which was 85% higher than 495–3 (Fig. 1B). On this basis, *O. polymorpha* was engineered for overproducing FFA. It has been showed that deletion of fatty acyl-CoA synthetase (encoded by *FAA1* and *FAA4*) resulted high level production of FFA in *S. cerevisiae* (Scharnewski et al. 2008; Zhou et al. 2016). We here thus tried to disrupt fatty acyl-CoA synthetase gene *FAA1* in 495–3 by the CRISPR/Cas9 system, and subsequently *OpLEU2* was also supplemented to obtain strain FD09. *FAA1* deletion resulted 8.8 folds higher FFA production (674 mg/L) with no obvious effect on cell growth compared with the control strain FD07 (Fig. 1B, C), demonstrating that *FAA1* encoded the main fatty acyl-CoA synthetase in *O. polymorpha.* Interestingly, main five types of FFA were detected in the engineered strain, including palmitoleic acid (C16:1), palmitic acid (C16:0), linoleic acid (C18:2), oleic acid (C18:1), and stearic acid (C18:0) (Fig. 1C).

### Co-feed of glucose and xylose

**Fig. 2:**
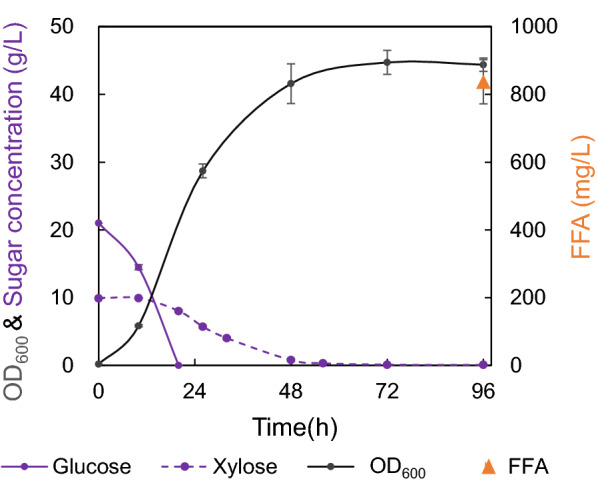
Co-feed of glucose and xylose for fatty acid over-production. Fermentation was carried out in minimal medium containing a mixture of 20 g/L glucose and 10 g/L xylose (20G + 10X) at 37 °C 220 rpm for 96 h. Samples were taken to analyze the cell growth, FFA titer and sugar concentration. Data are presented as mean ± s.e.m. (n = 3 biologically independent samples)

Lignocellulose is an inexpensive, abundant and sustainable feedstock, which mainly contains glucose and xylose with an approximate ratio of 2:1 and has been generally considered as a promising feedstock for bio-refinery. In this case, 20 g/L glucose and 10 g/L xylose were applied to simulate lignocellulosic hydrolysates for FFA production in engineered *O. polymorpha*. The prototrophic strain FD09 co-utilized glucose and xylose with FFA titer of 837 mg/L and final OD_600_ of 44.7 (Fig. 2). Glucose was quickly consumed within 24 h, and then xylose was exhausted in the next 24 h (Fig. 2). These results suggested glucose metabolism brought catabolite repression on xylose metabolism in *O. polymorpha*.

### Co-feed of methanol and glucose

**Fig. 3:**
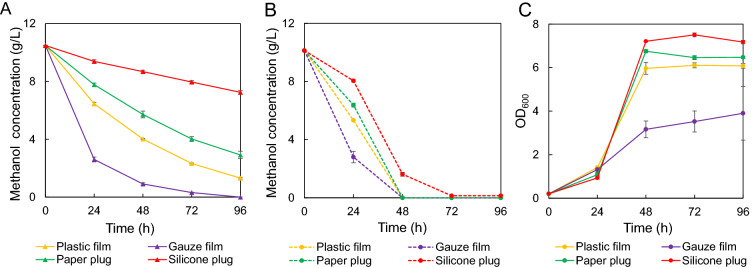
Effects of different types of plugs on methanol volatilization and strain growth. **A** Effect of different types of plugs on methanol volatilization without cell growth. **B** Methanol concentration of strain 495–3 in four types of plugs: Plastic film, Gauze film, Paper plug, silicone plug. **C** Growth of strain 495–3 in different types of plugs. The fermentation was carried out in minimal medium containing 10 g/L methanol at 37 °C 220 rpm for 96 h. Data are presented as mean ± s.e.m. (n = 3 biologically independent samples)

Methanol represents a promising low-cost feedstock to produce FFA (Duan et al. 2018). In addition, methanol can also promote the pretreatment of lignocellulose (Warner et al. 2014). Therefore, we evaluate the methanol utilization for FFA production. Firstly, methanol volatilization was observed during the fermentation, which seriously prevented the high FFA yields. We thus tried to prevent methanol volatilization by comparing different types of plugs including plastic film, gauze film, paper plug and silicone plug (Fig. 3). In regarding of loading cells or without cells, methanol volatilized fastest in shake flask with gauze film, followed by that of plastic film and paper plug. Silicone plug had the best performance in preventing methanol volatilization (Fig. 3A, B). Consistently, strain 495–3 had the highest final OD_600_ of 7.5 when cultivating in shake flasks with silicone plug (containing 10 g/L methanol), which was 11%, 23%, 92% higher than those of paper plug, plastic film and gauze film, respectively (Fig. 3C). Thus, silicone plug was used for shake flask fermentation.

**Fig. 4:**
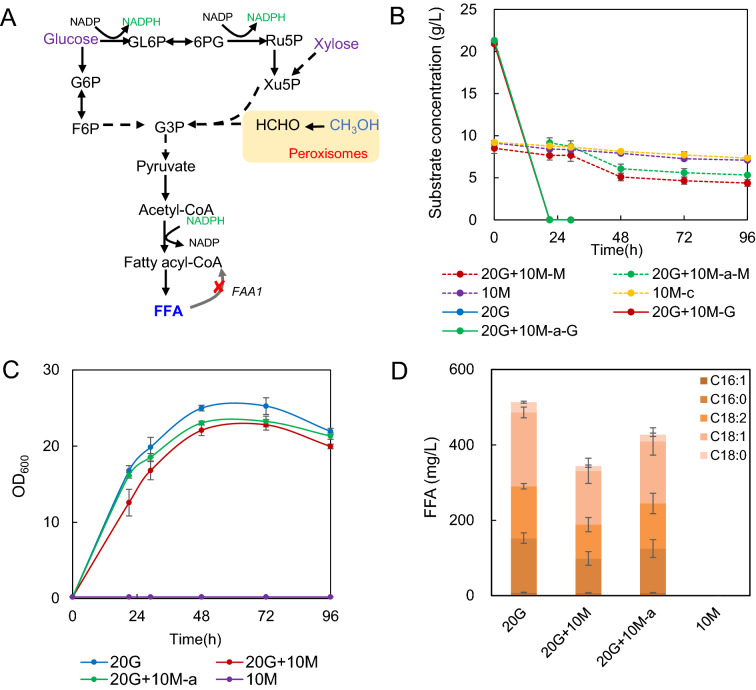
Co-feed of glucose and methanol for FFA production in strain FD09. **A** Schematic diagram of glucose and methanol metabolism in *O. polymorpha*. Supplemented glucose will provide extra NADPH for both cell growth and FFA production. **B** Concentration of carbon sources (-M: methanol concentration, -G: glucose concentration, 10 M-c: control of methanol volatilization without inoculation) in different carbon sources. 20G: 20 g/L glucose, 10 M: 10 g/L methanol, 20G + 10 M: 20 g/L glucose and 10 g/L methanol, 20G + 10 M-a: 20 g/L glucose with 10 g/L methanol supplemented at 24 h. **C** Growth curve of FD09 in different carbon sources. **D** FFA titer of FD09 in different carbon sources. All strains were cultivated in minimal medium with the specific substrates. Data are presented as mean ± s.e.m. (n = 3 biologically independent samples)

Surprisingly, our engineered FFA producing strain (*faa1*Δ) could not grow in minimum medium containing methanol as sole carbon source (Fig. 4C), which might be attributed to the limited supply of precursor xylulose-5-phophaste (Xu5P). We thus tried to supplement co-substrate glucose for promoting methanol utilization and FFA production, since glucose metabolism was supposed to increase the supply of Xu5P (Fig. 4A). Methanol was slightly used in glucose and methanol mixed medium (Fig. 4B), the cell growth was slightly retarded compared with that of sole glucose medium (Fig. 4C), which might be attributed to the methanol toxicity. Correspondingly, FFA titers in mixed substrates were 33% (methanol was initial added) and 17% (methanol was added at 24 h) lower in mixture of glucose and methanol compared with that of glucose medium, respectively (Fig. 4D). These data suggested that glucose didn’t significantly promote methanol utilization, which might be explained that the gene promoters of methanol metabolism were severely repressed by glucose (Zhai et al. 2021).

### Co-feed of methanol and xylose

**Fig. 5:**
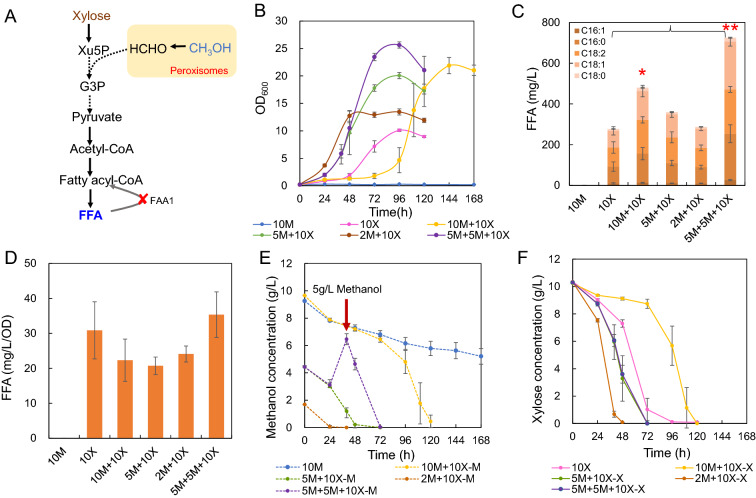
Co-feed of xylose and methanol for FFA production in strain FD09. **A** Schematic diagram of xylose and methanol metabolism in *O. polymorpha*. Supplemented xylose will increase Xu5P supply via xylose metabolism for both cell growth and FFA production. **B** Growth curves of strain FD09 in different carbon sources. 10 M: 10 g/L methanol, 10X: 10 g/L xylose, 10 M/5 M/2 M + 10X: 10 / 5 / 2 g/L methanol and 10 g/L xylose, 5 M + 5 M + 10X: Initial adding 5 g/L methanol and 10 g/L xylose and then supplementing 5 g/L methanol at 48 h. **C** FFA titers of strains cultivated in minimal media containing above substrates. Red asterisks indicate statistical significance as determined by paired *t* test (*P < 0.05; **P < 0.01). **D** Specific FFA production rate of strains cultivated in minimal media containing above substrates. **E** Methanol concentration. **F** Xylose concentration. Data are presented as mean ± s.e.m. (n = 4 biologically independent samples)

Methanol utilization pathway requires Xu5P for methanol assimilation (Fig. 5A), suggesting that xylose could promote methanol utilization via the enhancement of Xu5P supply. Therefore, a mixed xylose and methanol were used as substrates to evaluate the cell growth and FFA production of strain FD09. Supplementation of 10 g/L xylose (10 M + 10X) significantly enhanced methanol utilization and cell growth (Fig. 5B, E). The cells cultured in the mixture of xylose and methanol (10 M + 10X) had much higher cell biomass (OD_600_ 21.9 vs 10.1, Fig. 5B) which also had much higher FFA titer (480 vs 277 mg/L, Fig. 5C) compared with that of 10 g/L xylose (10X). These results suggested that there was a synergy between xylose and methanol metabolism (Fig. 5A). We observed there was a longer lag phase when cultivating the strain in 10 g/L methanol and xylose compared with sole xylose (Fig. 5B), which might be attributed to methanol toxicity. Lower methanol concentration indeed greatly decreased the lag phase time compared with that of 10 g/L methanol (Fig. 5B) and slightly improved FFA production than that of 10 g/L xylose (Fig. 5C). To balance the production and methanol toxicity, we applied a two-stage strategy with adding 5 g/L methanol to 10 g/L xylose at the initial cultivation and adding another 5 g/L methanol after 48 h (5 M + 5 M + 10X). These two stages of methanol supplementation greatly reduced the lag phase time and had the highest biomass (OD_600_ 25.6) and FFA production (725 mg/L) (Fig. 5B, C). The synergy between methanol and xylose utilization was observed within a suitable concentration range (Fig. 5E, F), which suggested that xylose could promote methanol assimilation and FFA production in *faa1*Δ strain. When utilizing methanol as co-substrates, the specific FFA titer (mg/L/OD) was slightly decreased (Fig. 5D), which might be attributed to methanol toxicity.

### Co-feed of glucose, xylose and methanol

**Fig. 6:**
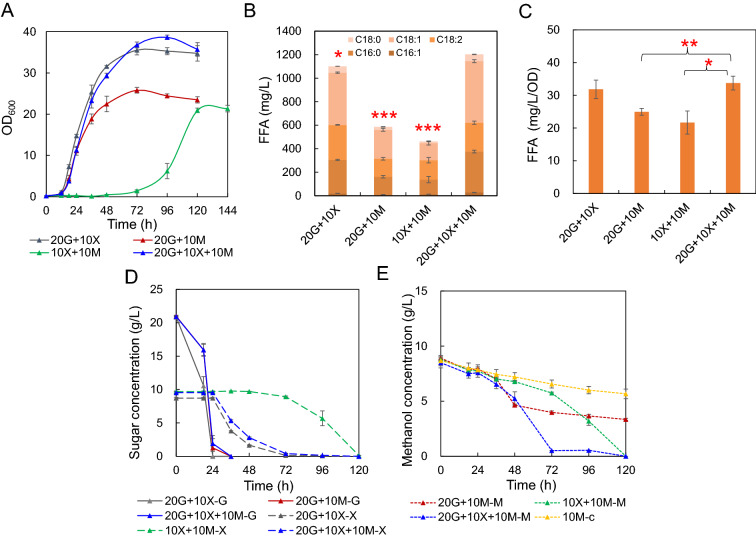
Co-feed of glucose, xylose and methanol for FFA production in strain FD09. **A** Growth curve of strain FD09 in different carbon sources. 20G + 10X: 20 g/L glucose and 10 g/L xylose, 20G + 10 M: 20 g/L glucose and 10 g/L methanol, 10X + 10 M: 10 g/L xylose and 10 g/L methanol, 20G + 10X + 10 M: 20 g/L glucose, 10 g/L xylose and 10 g/L methanol; Strains were cultivated in minimal media containing above substrates, and samples were taken to measure FFA titers. **B** FFA titers of strains cultivated in minimal media containing above substrates. Red asterisks indicate statistical significance as determined by paired *t* test (*P < 0.05; ***P < 0.001). **C** Specific FFA production rate of strains cultivated in minimal media containing above substrates. Red asterisks indicate statistical significance as determined by paired *t* test (*P < 0.05; **P < 0.01). **D** Sugar concentration ( G: glucose concentration, X: xylose concentration). **E** Methanol concentration (M: methanol concentration, 10 M-c: control of methanol volatilization). Data are presented as mean ± s.e.m. (n = 3 biologically independent samples)

Though glucose metabolism brought catabolite repression in *O. polymorpha* (Fig. 4), we explored the possible co-feed of methanol with glucose and xylose, which may simultaneously exist in real lignocellulosic hydrolysates (Warner et al. 2014). 20 g/L glucose, 10 g/L xylose and 10 g/L methanol was mixed for FFA production in strain FD09. Compared with co-feed of two specific substrates, the co-feed of glucose, xylose and methanol (20G + 10X + 10 M) achieved the highest maximum OD_600_, which was 5% higher (**P < 0.01) than that of glucose and xylose mixture (20G + 10X), 50% higher than that of glucose and methanol mixture (20G + 10 M), and 81% higher than that of xylose and methanol mixture (10X + 10 M), respectively (Fig. 6A). The FFA production of 20G + 10X + 10 M (1.2 g/L) was 9% higher (*P < 0.05) than that of 20G + 10X, and obviously higher (105% and 160%, respectively) than those of 20G + 10 M and 10X + 10 M (Fig. 6B). After consumption of glucose, methanol and xylose could be consumed simultaneously, and methanol was completely consumed at 72 h (Fig. 6D, E). Similar to results in Fig. 5D, methanol slightly reduced specific FFA production (mg/L/OD, Fig. 6C). Interestingly, strain cultivation in 20G + 10X + 10 M medium had the highest methanol consumption rate, which indicated that *O. polymorpha* possessed the greatest potential for co-feed of methanol and lignocellulosic hydrolysates.

## Discussion

Renewable feedstocks are required to satisfy the increasing demands of bio-refinery and contribute to carbon neutrality goal. Lignocellulosic biomass is abundant and renewable resources, thus becoming a promising feedstock for bio-manufacturing. In addition, methanol, which can be produced from coal, natural gas, and even CO_2_, has been considered as a promising feedstock (Shih et al. 2018). Methanol biotransformation by microorganism is a feasible way to utilize one carbon (C1) clean energy, which has a broad application prospect (Duan et al. 2018).

Though several chemicals were produced from methanol through engineered microbes (Yamada et al. 2019; Miao et al. 2021; Cai et al., 2022), we here observed that the engineering *O. polymorpha* for FFA production failed in growing in minimal methanol medium. Co-feeding xylose and methanol significantly enhanced methanol utilization and also behaved much better in cell growth and FFA production than that of sole xylose medium. This can be well explained that xylose can stimulate Xu5P cycle for methanol assimilation. Supplementing glucose brought strong repression on utilization of xylose and methanol. Alleviating the glucose repression should be helpful to enhance co-feed of xylose and methanol (Vasylyshyn et al. 2020). Anyway, we here found that methanol stimulated xylose utilization, which should be helpful for utilizing lignocellulosic hydrolysates, since methanol can enhance biomass pretreatment (Warner et al. 2014). In particularly, the sequential utilization of glucose and xylose was also observed in *O. polymorpha*. Enhancing xylose assimilation by engineering native xylose utilization pathways, introducing heterogenous xylose isomerase (XI) pathway, and alleviating xylose uptake, may be appropriate options to relieve glucose repression.

We here showed that disruption of fatty acyl-CoA synthase gene *FAA1* in *O. polymorpha* resulted in high-level production of FFA, which was higher than that of model microbial chassis cells (*E. coli* and *S. cerevisiae*) (Chen et al. 2020; Dai et al. 2017). This result showed great potential of *O. polymorpha* as a chassis cell for production of high-value chemicals. Currently, methylotrophic yeasts are mainly used for protein production (Ravin et al. 2013), which may be the limitation of available tools and genetic information (Gao and Zhou 2020). The development of genetic engineering platform and systems biology should pave the road for engineering *O. polymorpha* for overproducing a variety of chemicals other than fatty acids.

## Conclusions

In this study, the strain with FFA overproduction was obtained after deletion of fatty acyl-CoA synthetase gene *FAA1*. The engineered strain realized the co-feed of glucose, xylose and methanol, which lay a foundation for co-feeding of methanol and lignocellulosic hydrolysates to produce FFA and other high-value chemicals in *O. polymorpha.*

## Data Availability

The data sets in the current study are available from the corresponding author on reasonable request.

## Abbreviations

FFA: Free fatty acids
*FAA1*: Gene that encodes fatty acyl-CoA synthetase
*faa1*Δ: Gene that encodes fatty acyl-CoA synthetase was knocked out
α-IPM: α-Isopropyl malic acid
β-IPM: β-Isopropyl malic acid
α-KIC: α-Ketoisocaproic acid
HMG-CoA: Hydroxymeglutaryl-CoA
OD_600_: Optical density at wavelength 600 nm
GC: Gas chromatography
HPLC: High-performance liquid chromatography
